# Development of a robust daily soil temperature estimation in semi-arid continental climate using meteorological predictors based on computational intelligent paradigms

**DOI:** 10.1371/journal.pone.0293751

**Published:** 2023-12-27

**Authors:** Meysam Alizamir, Kaywan Othman Ahmed, Sungwon Kim, Salim Heddam, AliReza Docheshmeh Gorgij, Sun Woo Chang

**Affiliations:** 1 Department of Civil Engineering, Hamedan Branch, Islamic Azad University, Hamedan, Iran; 2 Department of Civil Engineering, Tishk International University—Sulaimani, Kurdistan Region, Iraq; 3 Department of Railroad Construction and Safety Engineering, Dongyang University, Yeongju, Republic of Korea; 4 Faculty of Science, Agronomy Department, Hydraulics Division, University 20 Août 1955 Skikda, Skikda, Algeri; 5 Faculty of Industry and Mining (Khash), University of Sistan and Baluchestan, Khash, Iran; 6 Department of Hydro Science and Engineering Research, Korea Institute of Civil Engineering and Building Technology, Goyang-si, Republic of Kore; King Fahd University of Petroleum and Minerals, SAUDI ARABIA

## Abstract

Changes in soil temperature (ST) play an important role in the main mechanisms within the soil, including biological and chemical activities. For instance, they affect the microbial community composition, the speed at which soil organic matter breaks down and becomes minerals. Moreover, the growth and physiological activity of plants are directly influenced by the ST. Additionally, ST indirectly affects plant growth by influencing the accessibility of nutrients in the soil. Therefore, designing an efficient tool for ST estimating at different depths is useful for soil studies by considering meteorological parameters as input parameters, maximal air temperature, minimal air temperature, maximal air relative humidity, minimal air relative humidity, precipitation, and wind speed. This investigation employed various statistical metrics to evaluate the efficacy of the implemented models. These metrics encompassed the correlation coefficient (r), root mean square error (RMSE), Nash-Sutcliffe (NS) efficiency, and mean absolute error (MAE). Hence, this study presented several artificial intelligence-based models, MLPANN, SVR, RFR, and GPR for building robust predictive tools for daily scale ST estimation at 05, 10, 20, 30, 50, and 100cm soil depths. The suggested models are evaluated at two meteorological stations (i.e., Sulaimani and Dukan) located in Kurdistan region, Iraq. Based on assessment of outcomes of this study, the suggested models exhibited exceptional predictive capabilities and comparison of the results showed that among the proposed frameworks, GPR yielded the best results for 05, 10, 20, and 100cm soil depths, with RMSE values of 1.814°C, 1.652°C, 1.773°C, and 2.891°C, respectively. Also, for 50cm soil depth, MLPANN performed the best with an RMSE of 2.289°C at Sulaimani station using the RMSE during the validation phase. Furthermore, GPR produced the most superior outcomes for 10cm, 30cm, and 50cm soil depths, with RMSE values of 1.753°C, 2.270°C, and 2.631°C, respectively. In addition, for 05cm soil depth, SVR achieved the highest level of performance with an RMSE of 1.950°C at Dukan station. The results obtained in this research confirmed that the suggested models have the potential to be effectively used as daily predictive tools at different stations and various depths.

## 1. Introduction

Soil temperature (ST) as a micro-meteorological parameter plays a crucial role in the agricultural water management, forests and deserts, geo-environmental processes, climatological and hydrological modeling, climate change, and solar energy studies [1–3]. Typically, ST can be regarded as an important parameter in determining the effectiveness of agricultural activities since it significantly influences processes such as root conditions, evapotranspiration, evaporation, and microorganism activities [4–6]. ST parameter is closely related to the soil heat flux within the energy equilibrium equation of the surface of the Earth [7,8]. Also, It plays a significant role in governing numerous physical, chemical, and biological activities taking place within the soil [9–11]. There are two different ways to estimate soil temperature, and they involve either analyzing soil heat flow and energy balance [12] or using correlations with related variables [13]. While the previously suggested methods may yield precise forecasts for a thoroughly assessed location, its applicability across various terrains is challenging due to a lack of adequate data to compute heat transfer equations or to find statistical relationship [14].

Nowadays, the monitoring and comprehension of soil conditions have experienced a noteworthy enhancement through the utilization of modern techniques for measuring ST and moisture in situ [15–17]. These measurements hold critical importance across several domains, such as agriculture, water resource managment, environmental science, meteorology and climatology, and geotechnical engineering [17].

In the realm of ST measurement, portable digital soil thermometers have emerged as versatile tools that offer quick and precise readings at multiple depths [18]. These devices are frequently utilized for prompt on-site assessments. Conversely, ST probes provide a continuous monitoring capability and can be strategically positioned at specific depths for prolonged durations [19,20]. This characteristic renders them highly advantageous for applications in hydrology and agriculture. Furthermore, the integration of ST sensors into data logging systems facilitates the acquisition of continuous, real-time temperature data [19]. This integration empowers researchers to effectively investigate and analyze fluctuations of temperature over an extended period.

Various reliable methodologies exist for soil moisture measurement. For this purpose, Time Domain Reflectometry (TDR) and Frequency Domain Reflectometry (FDR) instruments utilize electromagnetic waves to ascertain soil moisture content [21–23]. TDR evaluates the duration required for electromagnetic pulses to reflect back from the soil, while FDR employs diverse frequencies [23]. Both techniques exhibit remarkable precision and find widespread application in different fields of study [22,23]. Furthermore, capacitance sensors represent an additional prominent option for continuous monitoring of soil moisture [24,25]. These sensors rely on alterations in electrical capacitance induced by fluctuations in water content within the soil [25].

Moreover, the utilization of soil moisture probes positioned at varying depths within the soil enables the acquisition of uninterrupted data [26]. These probes play a pivotal role in comprehending the spatial distribution of moisture throughout the soil profile and are commonly employed in precision agriculture practices [27]. Additionally, the advent of remote sensing techniques, including satellites and aerial frameworks equipped with specialized sensors, has brought about a revolutionary transformation in measurement of values of soil moisture and ST parameters [28,29].

In contrast, the basic emperical regression methodologies rely on a limited number of variables like air temperature and leaf area index. Moreover, there are different elements that can limit the direct ST measurement. For example, the ST measured at a specific depth might not accurately reflect the distribution of temperature in the soil, since temperatures can differ greatly at different depths [30]. Furthermore, the placement of temperature sensors in the soil can impact the precision of the recorded data. Also, The existence of plants or other barriers may impede the positioning of sensors and result in distorted measurements [30]. Also, in relation to in situ observations, there remains significant uncertainty attributable to instrument inaccuracies and spatial variations. Additionally, the installation of a dense observation network is both cost-prohibitive and impractical [30].

Numerous researchers have explored various analytical models to investigate ST dynamics. For example, Droulia et al. (2009) [31] devised an analytical model that builds upon the existing general formula by substituting the steady state ST with readily obtainable daily average temperatures. To investigate the potential for reducing data requirements, they implemented various subsets of ST during the model development process. Upon comparing the model results with observational data, it was found that the suggested model provides a reasonably accurate approximation of the observed sequences of hourly ST. Zhang et al. (2021) [32] have introduced a novel approach for accurately predicting ST and the freezing front position. The model involves the development of a new mathematical structure derived from various model tests conducted under varous circumstances: sudden seepage, constant seepage, and no seepage. Additionally, a method based on regression analysis is employed to provide the coefficients within the equation. To validate the propsed model, it was checked by a traditional analytical method using data from both model tests and a real case study. The findings confirmed that the model exhibits superior stability and practicality when compared to traditional methods, offering reliable estimations of actual ST.

While analytical methods have traditionally been employed for ST prediction, they possess inherent limitations [33,34]. A major limitation is that these methods frequently rely on assumptions concerning the composition of soil, thermal characteristics, and boundary conditions that may not accurately reflect real-world scenarios [14]. In addition, analytical methods often rest on simplified mathematical approaches that suppose uniformity in characteristics of soil and neglect variables like moisture of soil, heterogeneity of soil, and the existence of vegetation [14]. Therefore, such simplifications can result in substantial inaccuracies when predicting ST, especially in intricate soil ecosystems [33,34]. Finally, it is of utmost importance to recognize these constraints while utilizing analytical paradigms and explore alternative tools like artificial intelligence models. This can help in predicting soil temperatures that are more precise and dependable.

ST is influenced by numerous elements. These elements affect the heat received at the surface, including solar radiation, crop coverage, pressure of air, color of soil, characteristics of soil heat, precipitation, organic content within the soil, and parameter of evaporation [35,36]. These various factors collectively play a role in determining the heat quantity that is provided to the soil surface. Moreover, the diffusion of temperature within the profile of the soil is affected by several factors, including soil moisture content and density [37].

For the past twenty years, artificial intelligence techniques have been utilized successfully in various engineering applications, particularly for water resource problems and hydrological studies and these methods have demonstrated remarkable efficacy and precision [38,39]. Delbari et al. (2019) [40] examined the effectiveness of a model based on support vector regression (SVR) in approximating the daily soil temperature at various depths (10, 30, and 100cm) under various weather patterns. In this study, different climatic parameters were applied as the input variables. The researchers compared these results with those obtained using the traditional multiple linear regression (MLR) method and confirmed that SVR outperformed MLR in accurately predicting ST at deeper layers. Feng et al. (2019) [19] utilized four distinct machine learning tools to simulate ST at depths of 02, 05, 10, and 20cm. The findings indicated that among the models tested, ELM demonstrated the highest level of performance across different time intervals for all depths. Additionally, they suggested that combining ELM with other optimization algorithms could enhance the ST estimation at various depths.

A comparison was carried out by Alizamir et al. (2020) [41] using four different machine learning methods for estimating monthly soil temperatures. These methods included extreme learning machine (ELM), group method of data handling (GMDH), classification and regression trees (CART), and artificial neural networks (ANN). They utilized monthly climatic data as inputs for their models. Overall, the findings revealed that ELM outperformed the other techniques in accurately modeling monthly ST. Li et al. (2020) [42] introduced an innovative approach to predict ST at various depths on an hourly basis. Their method involved utilizing a deep bidirectional long short-term memory network (BiLSTM), which integrated multiple meteorological factors as predictor parameters. To demonstrate the superiority of their approach, they compared it against six benchmark algorithms: LSTM, BiLSTM, deep neural network (DNN) from the deep learning (DL) approaches, as well as random forest (RF), linear regression, and support vector regression (SVR), from conventional models.

Penghui et al. (2020) [43] introduced a novel approach called ANFIS-mSG, which combines an ANFIS approach with optimization techniques using the mutation salp swarm algorithm and grasshopper optimization algorithm. This model was utilized to predict daily ST based on climatic data. The outcomes were compared to several models, including standalone ANFIS and various hybridized types of ANFIS models.

Bayatvarkeshi et al. (2021) [44] conducted a research in Iran using data collected from 12 locations between 2000 and 2010. In the initial phase of the study, they examined the impact of variation of climate on ST fluctuations at various depths (05, 10, 20, 30, 50, and 100cm). They used temperature of air as the independent variable and ST as the dependent parameter. By evaluation of the results of approaches for ST estimation, the findings suggested that the wavelet transformation combined with CANFIS (WCANFIS) model demonstrated a high level of predictive capability. Finally, the study indicates that the WCANFIS model has significant potential for estimating ST, particularly in diverse climatic regions.

Alizamir et al. (2021) [45] evaluated the performance of a new Deep ESN model with three classical approaches in predicting ST at depths of 10cm and 20cm. They created the Deep ESN model by combining various important daily hydro-meteorological data in six various scenarios from input parameters. To assess the accuracy of the ST models, they used three specific measures. The evaluation results demonstrated that the Deep ESN model showed the best performance compared to the classical methods, achieving a significant reduction of 30% to 60% in the RMSE accuracy indicator compared to the traditional models at both studied locations.

Hao et al. (2021) [46] introduced a novel approach termed EEMD-CNN, which combines ensemble empirical mode decomposition with a convolutional neural network. The objective of this model was to estimate ST at depths ranging from 05cm to 30cm. In order to assess the effectiveness of their suggested model, they compared it against three other models: persistence forecast (PF), backpropagation neural network, and LSTM.

Malik et al. (2022) [47] investigated the prediction of daily ST at different depths. They employed several hybrid strategies by combining SVM, MLP, and ANFIS by slime mould algorithm (SMA), particle swarm optimization (PSO), and spotted hyena optimizer (SHO). By considering different input variables derived from daily meteorological parameters, five scenarios were created. The optimal scenario was determined through the gamma test (GT). The performance of proposed integrative models was assessed through statistical indicators and visual interpretation. The findings revealed that the SVM-SMA model exhibited superior estimation precision in comparison with the other approaches for soil depths of 05cm, 15cm, and 30cm.

Imanian et al. (2022) [48] thoroughly evaluate the effectiveness of various AI methods in predicting ST parameter. They considered different approaches, including both traditional regression techniques and more advanced methods such as deep learning. Multiple variables related to the land and atmosphere are used as inputs for the proposed paradigms. Through a sensitivity analysis, the significance of each climate variable was determined, leading to a reduction in the number of input variables from 8 to 7. The findings of this analysis demonstrated that air temperature and solar radiation play a crucial role in ST estimation, while precipitation can be disregarded. Comparing the AI models confirmed that deep learning achieves the highest performance, with an R-squared value of 0.980 and an NRMSE of 2.237%. Following closely behind is the multi-layer perceptron model, which attains an R-squared value of 0.980 and an NRMSE of 2.266%.

Farhangmehr et al. (2023) [49] devised a 1D convolutional neural network (CNN) model to forecast hourly soil temperature at a depth of 0-7cm. The model was trained using eight hourly climatic features spanning an entire year. Comparative analysis was conducted against a multilayer perceptron (MLP) model using diverse evaluation metrics. A sensitivity analysis revealed that air temperature exerted the most significant influence on soil temperature prediction, while surface thermal radiation had the least impact. The 1D convolutional model exhibited superior performance to the MLP model, particularly under normal and hot weather conditions. The study successfully showcased the capacity of this model to accurately forecast daily maximum soil temperature.

Chawang et al. (2023) [50] conducted an evaluation of the Noah land surface model’s performance in estimating soil moisture (SM) and soil temperature (ST) across India. The study utilized 3-hourly data at resolutions of 5km and 10km. Various precipitation inputs, including CHIRPS, GDAS, and IMERG, were considered, with CHIRPS yielding the best results at 5km resolution, while IMERG performed optimally at 10km resolution. Notably, the inclusion of a dynamic Greenness Vegetation Fraction in conjunction with IMERG enhanced the accuracy of SM and ST by up to 25.21% and 8.36°, respectively. The model exhibited improved performance over clay, loam, and sandy clay loam soils, which encompass approximately 67% of India’s land area. At 10km resolution, the model attained surface SM accuracy of 0.095 m3/m3 and ST accuracy of 4.22 K. Evaluation metrics demonstrated strong correlation, low root mean square error, and minimal bias when compared to satellite SM data. These findings highlight the potential of land surface models in estimating SM and ST across India.

In earlier surveys, a restricted number of climatic factors were typically utilized. However, in the present study, a diverse array of weather parameters was applied. While numerous investigations have implemented artificial intelligence algorithms, they mostly concentrated on a limited set of weather variables, primarily air temperature. It is important to note that there are numerous other weather data that influence ST at different depths.

The major objective of this study is to implement several efficient models for estimating soil temperature in semi-arid continental climate. Therefore, this paper utilizes artificial intelligence models on two distinct stations to assess their ability to adapt and perform well across various levels of data complexity. The recommended methods are developed by considering various relevant weather variables over a specific timeframe that aligns with the desired soil temperature time series at Sulaimani and Dukan stations, Kurdistan region, Iraq. Moreover, a thorough analysis and evaluation of the modeling are conducted to ensure their effectiveness and applicability using several metrics for performance evaluation. This study explores the first time application of different artificial intelligence models including MLPNN, SVR, RFR, and GPR methods to estimate ST using diverse climatic data at Dukan and Sulaimani stations in Iraq. These innovative techniques demonstrate the ability to accurately estimate ST profiles under different climatic conditions. By incorporating multiple climatic variables such as air temperature, precipitation, humidity, and wind speed, these methodologies provide comprehensive insights into the dynamics of soil thermal behavior. The results enhance our understanding of the intricate relationships between climatic factors and ST, facilitating improved precision in agricultural planning, environmental monitoring, and assessment of climate change impacts.

The structure of this paper is as follows: Section 2 provides a detailed account of the data utilized in the current study, along with an explanation of the mathematical basis for the machine learning models employed. In Section 3, how models are evaluated is presented. Section 4 of the study showcases the outcomes obtained from the proposed models, along with a thorough evaluation of their effectiveness. Additionally, an in-depth analysis and discussion regarding these findings is provided in section 5. In the end, Section 6 encompasses the presentation of conclusions of this study. To the best of the authors’ knowledge, this study is the first to apply several artificial intelligence models in estimating soil temperature by considering different climatic time series at Sulaimani and Dukan stations, Kurdistan region, Iraq.

## 2. Methodology and model development

In the present study, daily meteorological data were used to estimate soil temperature in two different stations of Kurdistan region, Iraq. Four machine learning methods, MLPANN, SVR, RFR, and GPR were used to estimate soil temperature time series. Moreover, in this study, maximal air temperature, minimal air temperature, maximal air relative humidity, minimal air relative humidity, precipitation, wind speed were applied as predictor parameters.

### 2.1 Study area and data used description

**Fig 1:**
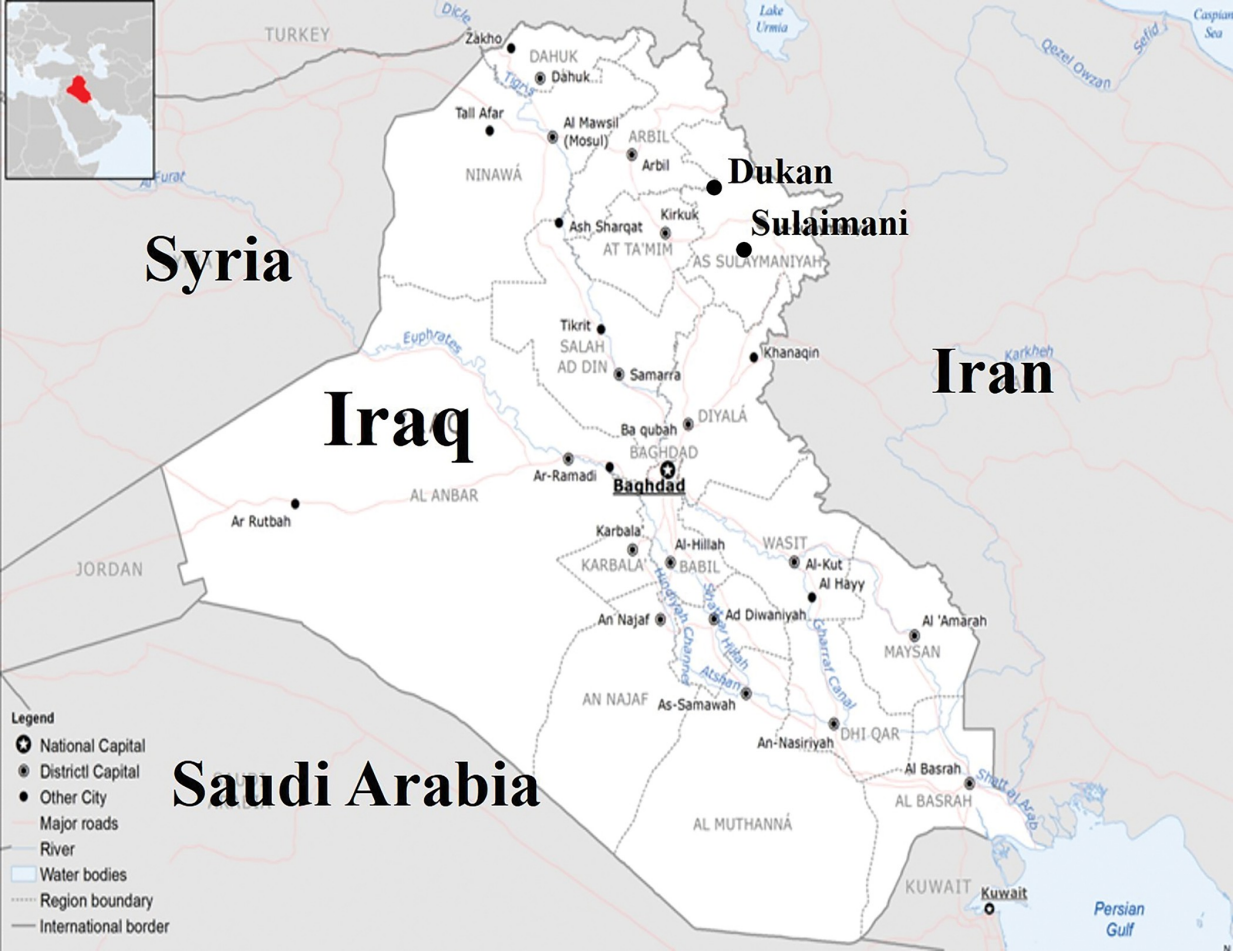
Map showing the location of Sulaimani and Dukan stations, Iraq (https://commons.wikimedia.org/wiki/File:Iraq_Base_Map.png).

In this research, the effectiveness of proposed artificial intelligence models was evaluated at Sulaimani and Duakan stations, Kurdistan region, Iraq (Fig 1). Tables 1 and 2 present the statistical features of the dataset utilized in this research, including mean (X_mean_), maximum (X_max_), minimum (X_min_), standard deviation (S_x_), and coefficient of variation (C_v_) of maximal air temperature (T_max_), minimal air temperature (T_min_), maximal air relative humidity (H_max_), minimal air relative humidity (H_min_), precipitation (P), wind speed (U_2_), and soil temperature (ST) based on different soil depths (i.e., ST-05, ST-10, ST-20, ST-50, and ST-100) at Sulaimani and Duakan stations. It can be judged from Table 1 that the standard deviation (S_x_) for parameters of air relative humidity (H_max_ and H_min_) presented higher values compared to other meteorological parameters. Also, T_max_ gave more extreme temperature than 46°C at Sulaimani station. It can be found from Table 2 that the T_max_ supplied more severe temperature over 46°C at Dukan station. In addition, the standard deviations of air relative humidity parameters supported higher outputs compared to other meteorological parameters. For this research, the data were split into 80% for training and 20% for testing to develop artificial intelligence models.

**Table 1:** Summary statistics of meteorological parameters and soil temperature at Sulaimani Station.

Parameters`	Depth (cm)	Unit	X_mean_	X_max_	X_min_	S_x_	C_v_
*T*_*max*_	**-**	°C	24.097	46.200	0.900	11.025	0.458
*T*_*min*_	**-**	°C	13.461	39.000	-5.900	9.083	0.675
*H*_*max*_	**-**	%	63.383	100.000	10.000	23.125	0.365
*H*_*min*_	**-**	%	35.931	97.000	6.000	21.548	0.598
*U*_*2*_	**-**	m/s	1.292	30.000	0.000	1.466	1.134
*P*	**-**	mm	2.653	131.800	0.000	8.578	3.233
ST-05	05	°C	18.509	42.700	-8.000	10.661	0.576
ST-10	10	°C	18.384	39.700	1.600	10.098	0.549
ST-20	20	°C	18.134	37.800	1.900	9.671	0.533
ST-50	50	°C	18.868	34.800	1.500	8.464	0.449
ST-100	100	°C	19.299	31.300	7.700	6.900	0.358

[Abbreviations: X_mean_, mean; X_max_, maximum; X_min_, minimum; S_x_, standard deviation; C_v_, coefficient of variation; *T*_*max*_: Maximal air temperature, *T*_*min*_: Minimal air temperature, *H*_*max*_: Maximal air relative humidity, *H*_*min*_: Minimal air relative humidity, *P*: Precipitation, U_2_: Wind speed, ST: Soil temperatures at 05, 10, 20, 50 and 100 cm, respectively.

**Table 2:** Summary statistics of meteorological parameters and soil temperature at Dukan station.

Parameters	Depth (cm)	Unit	X_mean_	X_max_	X_min_	S_x_	C_v_
*T*_*max*_	**-**	°C	25.383	48.000	1.000	12.091	0.476
*T*_*min*_	**-**	°C	15.400	34.000	-4.000	9.254	0.601
*H*_*max*_	**-**	%	66.325	100.000	18.000	19.250	0.290
*H*_*min*_	**-**	%	32.118	95.000	0.000	19.614	0.611
*U*_*2*_	**-**	m/s	2.164	11.550	0.000	1.344	0.621
*P*	**-**	mm	1.633	67.800	0.000	6.118	3.747
ST-05	05	°C	17.621	38.000	-2.000	10.437	0.592
ST-10	10	°C	18.801	37.000	-5.000	9.984	0.531
ST-30	30	°C	21.215	42.000	-4.000	9.508	0.448
ST-50	50	°C	22.102	37.000	6.000	8.603	0.389

[Abbreviations: X_mean_, mean; X_max_, maximum; X_min_, minimum; S_x_, standard deviation; C_v_, coefficient of variation; *T*_*max*_: Maximal air temperature, *T*_*min*_: Minimal air temperature, *H*_*max*_: Maximal air relative humidity, *H*_*min*_: Minimal air relative humidity, *P*: Precipitation, U_2_: Wind speed, ST: Soil temperature at 05, 10, 30 and 50 cm, respectively.

As mentioned, In order to develop and evaluate artificial intelligence techniques for ST estimation utilizing various climatic data, the Duakan and Sulaimani stations were selected as case study sites due to their semi-arid continental climate. These stations offer distinct solar radiation, air temperature, humidity, wind speed, and rainfall patterns, providing diverse conditions for the construction and assessment of ST estimation models. Furthermore, long-term monitoring networks have provided high-quality ST measurements at different depths for both stations. By constructing estimation models using data from these climatically contrasting regions, the objective is to establish efficient models capable of precisely predicting ST across a wide range of surface weather conditions. The evaluation of these models at the Duakan and Sulaimani stations will not only appraise their achievement in various climate regimes but also explore their potential widespread validity for global soil temperature estimation utilizing readily available climatic data.

Due to climate of Iraq which is characterized by high temperatures, assessing the soil temperature holds immense significance owing to its substantial influence on agricultural yield and the development of plants. By keeping track of the ST, farmers and agricultural professionals are able to gather valuable information to guide them in making well-informed choices regarding when to plant their crops, how to efficiently irrigate, and which types of crops are best suited for their specific conditions at the Dukan and Sulaimani stations. In other words, having this knowledge enables farmers in Iraq to improve their agricultural methods, which in turn can boost food production and security.

### 2.2 Gaussian Process Regression (GPR)

Gaussian Process Regression is a non-parametric and a non-linear regression modelling method [51,52]. It produces a limited set of arbitrary variables. GPR applies non-parametric Bayesian modelling, which contemplates the variance of the data set and the probability margin maximum in the training set, utilizing a scaled anisotropic Gaussian kernel function. GPR is a kind of supervised learning method, and permits to identify the significant features of the input variables [53]. Beside the assessing the relative contribution importance of applicable bands or parameters in forecasting process. GPR is advantageous because of its uncomplicated nature and precision [51]. Furthermore, GPR resists against the data overfitting [54]. Both the mean [*m(x)*] and covariance/kernel [*k (x*_*i*_, *x*_*j*_*)*] functions, generally applied to describe the GPR [55] as can be seen below:

$$f(x)∼GP[m(x).k(x_{i}.x_{j})]$$ (1)

The *x* in Eq (1) denotes each input vector. *m(x)* and *k (x*_*i*_, *x*_*j*_*)* can be stated as below, respectively.


m(x)=E[f(x)] (2)

$$k(x_{i},x_{j})=\mathrm{cov}[f(x_{i}),f(x_{j})]$$ (3)

**Fig 2:**
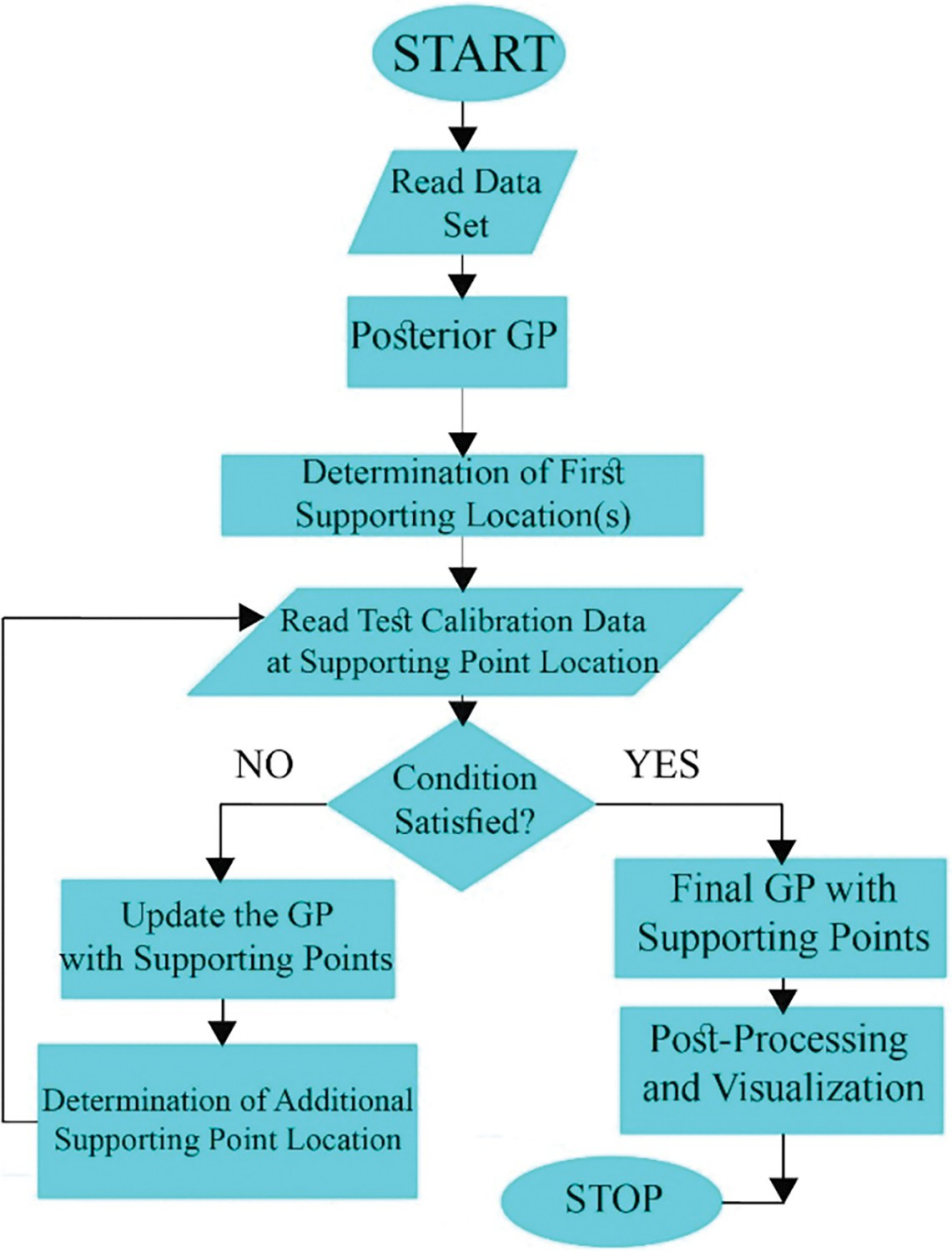
Gaussian Process Regression (GRP) architecture.

Fig 2 shows the schematic flowchart of GPR method.

### 2.3 Multi-layer perceptron artificial neural network (MLPANN)

**Fig 3:**
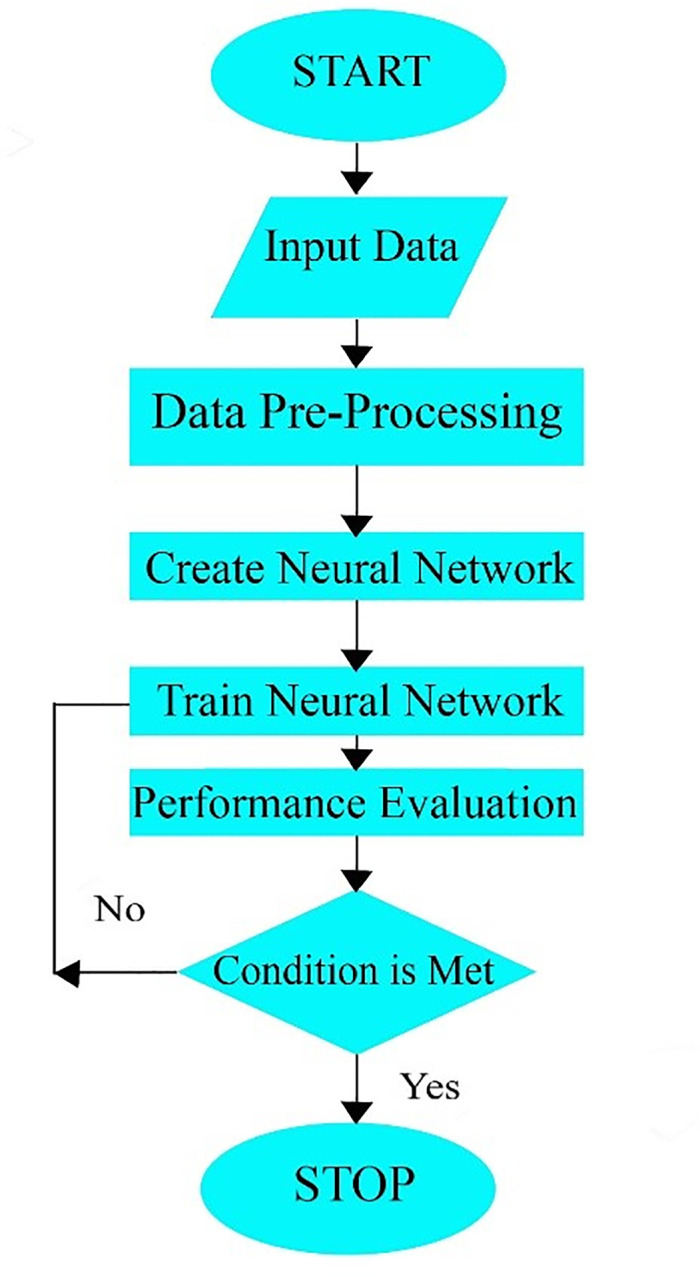
Multilayer perceptron neural network (MLPNN) architecture.

MLPANN rephrase this: Multilayer perceptrons (MLPs) are a highly effective type of supervised learning artificial neural network. They utilize the backpropagation algorithm to adjust weights and reduce error. It comprises of three diverse layers, called input, hidden, and output layer [56,57]. In this method, each separate neuron must be linked to all following layer neurons, while the neurons should be arranged in a one-directional procedure, ultimately [45,58]. Fig 3 represents the flowchart of MLPANN. Weights are being used in various layer connection to each other, range from -1 to 1. There are some nodes in MLPANN, which have two characters, named summation and activation [59]. Eq 4 can be utilized to calculate the input products, weights, and the model bias by employing a summation function:

$$S_{j}=\sum_{i=1}^{n}\omega_{ij}I_{i}+\beta_{j}$$ (4)

where *S*_*j*_ is the summation function, *n* represents the number of inputs, input variable *i* can be shown by *I*_*i*_, while *β*_*j*_ and *ω*_*ij*_ are bias term and connection weight, correspondingly. The activation function, subsequently, can be derived from the output of the summation equation. MLPANN has numerous forms of activation functions, which the utmost useful one is S-shaped curved sigmoid function [60], and can be clarified mathematically as below:

$$f_{j}(x)=\frac{1}{1+e^{-S_{j}}}$$ (5)

The last output of neuron j, eventually, could be calculated by means of below equation:

$$y_{i}=f_{i}(\sum_{i=1}^{n}\omega_{ij}I_{i}+\beta_{j})$$ (6)

In Fig 3 the different steps of MLPANN method can be seen via its flowchart.

### 2.4 Random Forest Regression (RFR)

Random Forest Regression is a method, merges the act of various Decision Tree (DT) algorithms in classification or prediction [61,62]. When RF receives (*x*) input vector, it constructs a number *K* regression trees and means the outcomes. The RF regression predictor can be stated mathematically as below:

$${\hat{f}}_{rf}^{K}(X)=\frac{1}{K}\sum_{k=1}^{K}T(X)$$ (7)

**Fig 4:**
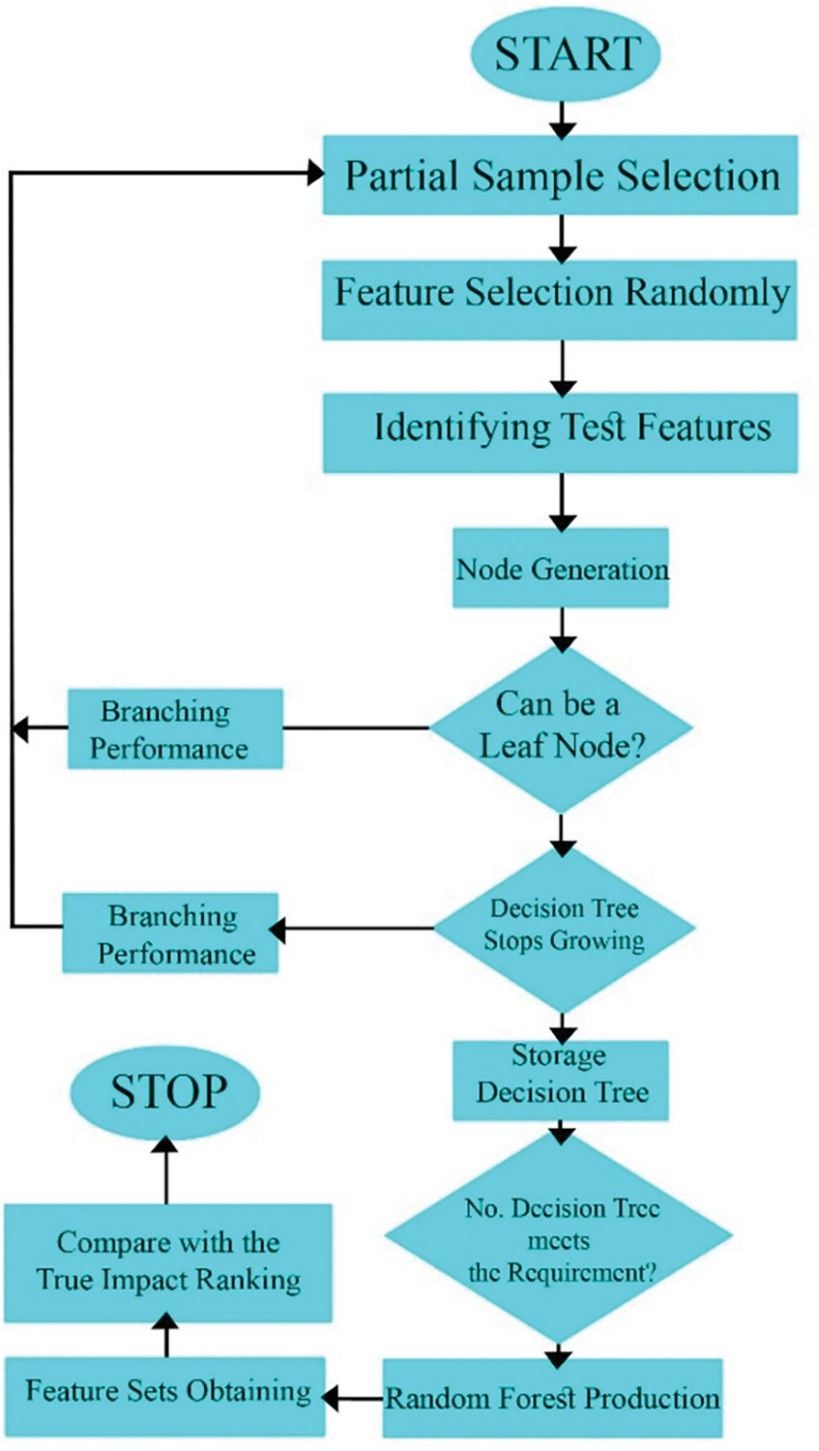
Random Forest Regression (RFR) architecture.

Bagging is a routine technique of RF to reduce the correlation among the different decision trees. Bagging is applied in training data making via accidental resampling of the original dataset by replacement procedure. Henceforth, some data might be utilized more than once in training phase, whereas others may never be used, and it could make better stability, which upsurges prediction accuracy consequently [63]. Conversely, during the tree growing, It makes use of the best characteristic/breaking point within a specific group of supporting traits. As a result, this might diminish the individual tree’s strength while concurrently weakening the interdependence among them, meanwhile, that diminishes the generalization error, subsequently [63]. Moreover, The specimens not selected for training the k^th^ tree in the bagging procedure are included as a fraction of an additional subset, known as the out-of-bag (OOB) samples. OOB fundamentals are applied by the k^th^ tree to assess the operation of model [64]. RF, in such cases, is able to compute an impartial estimate of generalization error without relying on the utilization of an external text data subset [63]. Fig 4 shows the different steps of RFR model via a schematic flowchart.

### 2.5 Support Vector Regression (SVR)

Support Vector Regression is a kind of prevalent machine learning method which has accurate outputs and low computation cost [65]. SVR is appropriate in treatment with insufficient dataset [66]. SVR can handle nonlinear relations perfectly, and shows its effectiveness in generalization process [67].

Support Vector Regression employs the utilization of kernel functions to execute a non-linear transformation technique, effectively mapping the initial input space into a novel hyperspace. In mathematical terms, the SVR can be represented as outlined below:

f(x)=ω.φ(x)+b (8)

where φ(x), ω and b represent non-linearly transformed training dataset, weight vectors that correspond to them, and the bias term, respectively. The coefficients (*ω* and *b*) are assessed via normalized risk function minimization, which can be represented as below:

$$R(C)=\frac{C}{n}{\sum_{i=1}^{n}L_{\varepsilon }(y_{i},f(x_{i}))+\frac{1}{2}\|\omega \|}^{2}$$ (9)

where:

$$L_{\varepsilon }(y_{i},f(x_{i}))=\{\begin{array}{c} |f(x_{i})-y_{i}|-\varepsilon ,for|f(x_{i})-y_{i}|\geq \varepsilon \\ 0,otherwise \end{array}$$ (10)

**Fig 5:**
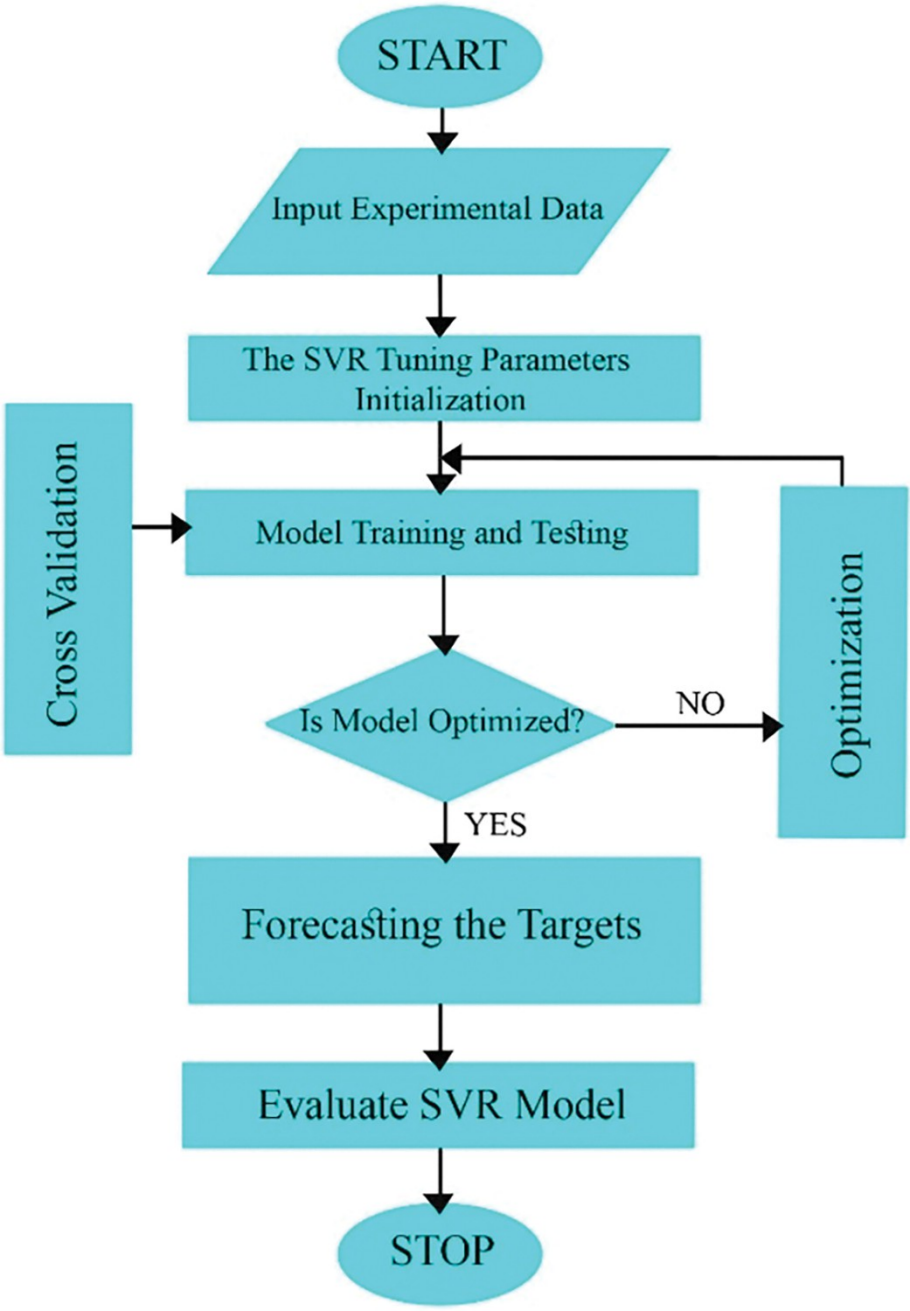
Support Vector Regression (SVR) architecture.

The following controlled equation can be express as below:

$$C\sum_{i=1}^{n}\left(\zeta_{i},\zeta_{i}^{*}\right)+\frac{1}{2}{\left\|\omega \right\|}^{2}$$ (11)

$$subject\{\begin{array}{c} y_{i}-(\omega \varphi (x_{i})+b_{i})\leq \varepsilon +\zeta_{i}\\ (\omega \varphi (x_{i})+b_{i})-y_{i}\leq \varepsilon +\zeta_{i}^{*}\\ \zeta_{i},\zeta_{i}^{*}\geq 0,i=1,\ldots ,n \end{array}$$ (12)

where $\frac{1}{2}{\left\|\omega \right\|}^{2}$ and $\frac{C}{n}\sum_{i=1}^{n}L_{\varepsilon }(y_{i},f(x_{i}))$ are the regularization term and empirical error, respectively. whereas *ζ*_*i*_ and $\zeta_{i}^{*}$ are slack variables, representing the positive and negative errors at the i_th_ point, correspondingly. *C* is the penalty factor, while ε is the loss function. The constrained optimization problem then could be answered by the Lagrangian and Karush-Kuhn-Tucker condition methods. Fig 5 shows the schematic flowchart of SVR model.

## 3.Performance evaluation

In this study, four artificial intelligence models were applied for soil temperature at different depths using several hydroclimatic data as input parameters. The outcomes of models were compared using the following statistical indices including correlation coefficient (R), root mean square error (RMSE), Nash-Sutcliffe (NS) efficiency, and mean absolute error (MAE):

$$R=\frac{\sum_{i=1}^{n}\left((ST{)}_{io}-(ST{)}_{io})((ST{)}_{ip}-(\bar{ST}{)}_{ip}\right)}{\sqrt{\sum_{i=1}^{n}((ST{)}_{io}-\bar{(ST{)}_{io}}{)}^{2}\sum_{i=1}^{n}((ST{)}_{ip}-\bar{(ST{)}_{ip}}{)}^{2}}}$$ (13)

$$RMSE=\sqrt{\frac{\sum_{i=1}^{n}{\left((ST{)}_{io}-(ST{)}_{ip}\right)}^{2}}{n}}$$ (14)

$$NS=1-\frac{\sum_{i=1}^{n}((ST{)}_{io}-(ST{)}_{ip}{)}^{2}}{\sum_{i=1}^{n}((ST{)}_{io}-\bar{(ST{)}_{io}}{)}^{2}}$$ (15)

$$MAE=\frac{1}{N}\sum_{i=1}^{N}\left|(ST{)}_{io}-(ST{)}_{ip}\right|$$ (16)

where n denotes the quantity of datasets. Also, (*ST*)_*io*_ and (*ST*)_*ip*_ indicate observed and estimated values for soil temperature parameter at different depths.

## 4. Results

This article utilized the diverse meteorological parameters for predicting soil temperature (ST) at Sulaimani and Dukan stations, Kurdistan region, Iraq. As described before, the assessment of employed ML models (MLPNN, SVR, RFR, and GPR) for predicting ST based on the different soil depths is the fundamental element of present research scheme. The predicting problem is focused on 05, 10, 20, 50, and 100cm at Sulaimani station, while it is concentrated on 05, 10, 20, and 50cm at Dukan station, respectively

### 4.1 Prediction of soil temperature based on different soil depths at Sulaimani station

#### 4.1.1 Application of MLPNN, SVR, RFR, and GRP models

The predictive issues of different MLPNN models utilized in this article based on four evaluation indices (MAE, RMSE, NSE, and R) are arranged in Table 3. The predictive assessments of MLPNN_10 (MAE = 1.371°C, RMSE = 1.768°C, NSE = 0.969, and R = 0.984) were more outstanding than those of MLPNN_05, MLPNN_20, MLPNN_50, and MLPNN_100 from the training dataset. Also, MLPNN_10 (MAE = 1.311°C, RMSE = 1.695°C, NSE = 0.972, and R = 0.986) performed more excellent prediction than MLPNN_05, MLPNN_20, MLPNN_50, and MLPNN_100 clearly from the validation dataset.

**Table 3:** Performances of different ML models for soil temperature modelling: Sulaimani station.

	Training				Validation			
Models	R	NSE	RMSE (°C)	MAE (°C)	R	NSE	RMSE (°C)	MAE (°C)
MLPNN_05	0.984	0.968	1.897	1.453	0.984	0.969	1.901	1.472
MLPNN_10	0.984	0.969	1.768	1.371	0.986	0.972	1.695	1.311
MLPNN_20	0.983	0.966	1.771	1.374	0.982	0.964	1.837	1.393
MLPNN_50	0.965	0.931	2.197	1.686	0.964	0.929	2.289	1.766
MLPNN_100	0.913	0.834	2.718	2.156	0.907	0.822	3.069	2.455
SVR_05	0.979	0.958	2.165	1.681	0.983	0.965	1.993	1.563
SVR_10	0.980	0.959	2.021	1.596	0.983	0.965	1.892	1.506
SVR_20	0.977	0.955	2.044	1.616	0.978	0.957	2.026	1.575
SVR_50	0.957	0.915	2.439	1.910	0.955	0.913	2.542	1.996
SVR_100	0.894	0.799	2.993	2.403	0.905	0.818	3.097	2.488
RFR_05	0.992	0.983	1.382	1.043	0.985	0.969	1.881	1.460
RFR_10	0.992	0.984	1.269	0.973	0.986	0.971	1.731	1.335
RFR_20	0.991	0.982	1.279	0.976	0.982	0.964	1.858	1.400
RFR_50	0.982	0.965	1.571	1.183	0.961	0.923	2.382	1.841
RFR_100	0.958	0.917	1.929	1.509	0.913	0.833	2.972	2.372
GPR_05	0.983	0.966	1.955	1.506	0.986	0.971	1.814	1.402
GPR_10	0.984	0.968	1.787	1.392	0.987	0.974	1.652	1.284
GPR_20	0.982	0.965	1.798	1.402	0.983	0.967	1.773	1.327
GPR_50	0.964	0.930	2.215	1.706	0.964	0.929	2.296	1.759
GPR_100	0.912	0.832	2.736	2.173	0.918	0.842	2.891	2.316

Conditional on the diverse SVR models, SVR_10 (MAE = 1.596°C, RMSE = 2.021°C, NSE = 0.959, and R = 0.980) supplied the best outputs compared to other SVR models from the training dataset. In addition, SVR_10 (MAE = 1.506°C, RMSE = 1.892°C, NSE = 0.965, and R = 0.983) gave the best outputs compared to other SVR models from the validation dataset.

Dependent on the various RFR models, RFR_10 (MAE = 0.973°C, RMSE = 1.269°C, NSE = 0.984, and R = 0.992) provided the topmost values compared to RFR_05, RFR_20, RFR_50, and RFR_100 from the training dataset. As well, RFR_10 (MAE = 1.335°C, RMSE = 1.731°C, NSE = 0.971, and R = 0.986) showed the topmost values compared to RFR_05, RFR_20, RFR_50, and RFR_100 from the validation dataset.

Relying on the numerous GPR models, GPR_10 (MAE = 1.392°C, RMSE = 1.787°C, NSE = 0.968, and R = 0.984) produced the highest values compared to GPR_05, GPR_20, GPR_50, and GPR_100 from the training dataset. Furthermore, GPR_10 (MAE = 1.284°C, RMSE = 1.652°C, NSE = 0.974, and R = 0.987) yielded the highest values compared to GPR_05, GPR_20, GPR_50, and GPR_100 from the validation dataset.

Comparing the models performance utilizing training and validation dataset based on RMSE values (°C) for MLPNN models, only MLPNN_10 utilizing validation dataset could overcome the model performance of training dataset. In case of SVR and GPR models, the predicted outputs performed by SVR and GPR models at 05, 10, and 20cm soil depths employing validation dataset could overwhelm the model performance of training dataset. Finally, no RFR models using validation dataset could win model performance of training dataset. Therefore, it can be judged that the model performance of training dataset was superior to that of validation dataset at Sulaimani station clearly.

**Fig 6:**
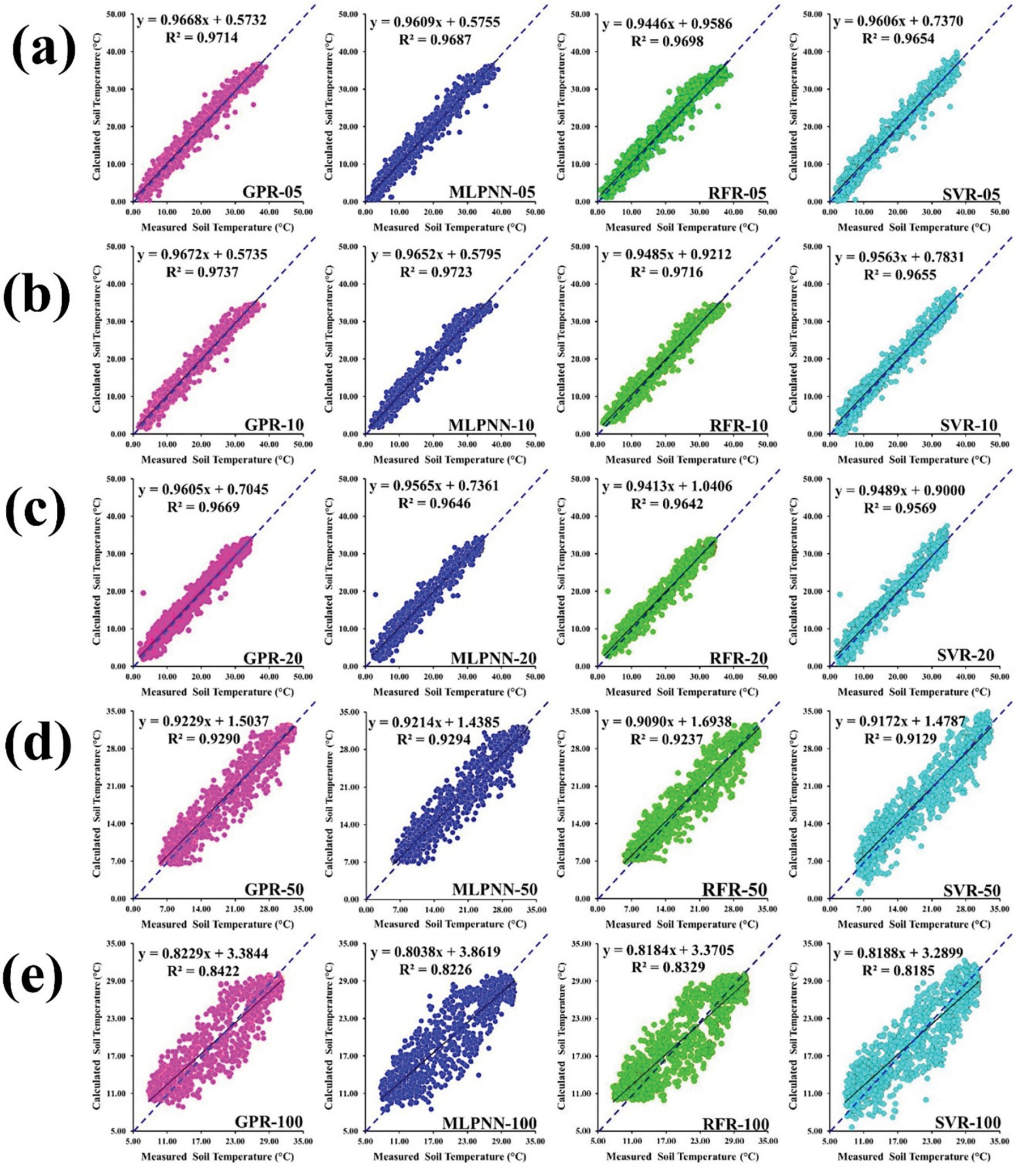
Scatterplot of measured versus predicted soil temperature of validation dataset for Sulaimani station and different soil depths: (a) 05cm, (b) 10cm, (c) 20cm, (d) 50cm, and (e) 100cm.

Fig 6(A)–6(E) illustrate the scatterplot of measured versus predicted soil temperature based on the different soil depths from the validation dataset at Sulaimani station. Each scatterplot consists of fitted line (solid), equal line (dotted), optimized regression equation, and determination coefficient, respectively. Relying on the values of determination coefficient, GPR_10 (R^2^ = 0.9737) furnished the maximum value compared to varied GPR models such as GPR_05, GPR_20, GPR_50, and GPR_100 from the validation dataset. Also, MLPNN_10 (R^2^ = 0.9723) recorded the best output compared to various MLPNN models including MLPNN_05, MLPNN_20, MLPNN_50, and MLPNN_100 from the validation dataset. In addition, RFR_10 (R^2^ = 0.9716) supplied the topmost output compared to diverse RFR models such as RFR_05, RFR_20, RFR_50, and RFR_100 from the validation dataset. As well, SVR_10 (R^2^ = 0.9655) provided the highest value compared to different SVR models including SVR_05, SVR_20, SVR_50, and SVR_100 from the validation dataset.

Based on the diverse models with 05cm soil depth, GPR_05 (R^2^ = 0.9714) showed the best output compared to different models including MLPNN_05, RFR_05, and SVR_05 from the validation dataset. In case of 10cm soil depth, GPR_10 (R^2^ = 0.9737) presented the highest value compared to various models including MLPNN_10, RFR_10 and SVR_10 from the validation dataset. From the Fig 6(C), GPR_20 (R^2^ = 0.9669) provided the topmost output compared to particular models such as MLPNN_20, RFR_20, and SVR_20 from the validation dataset. Considering 50cm soil depth, however, MLPNN_50 (R^2^ = 0.9294) furnished the maximum value compared to varied models such as GPR_50, RFR_50, and SVR_50 from the validation dataset. Recognizing 100cm soil depth, GPR_100 (R^2^ = 0.8422) yielded the top value compared to divergent models such as MLPNN_100, RFR_100, and SVR_100 from the validation dataset.

#### 4.1.2 Visual services for performances of machine learning models

**Fig 7:**
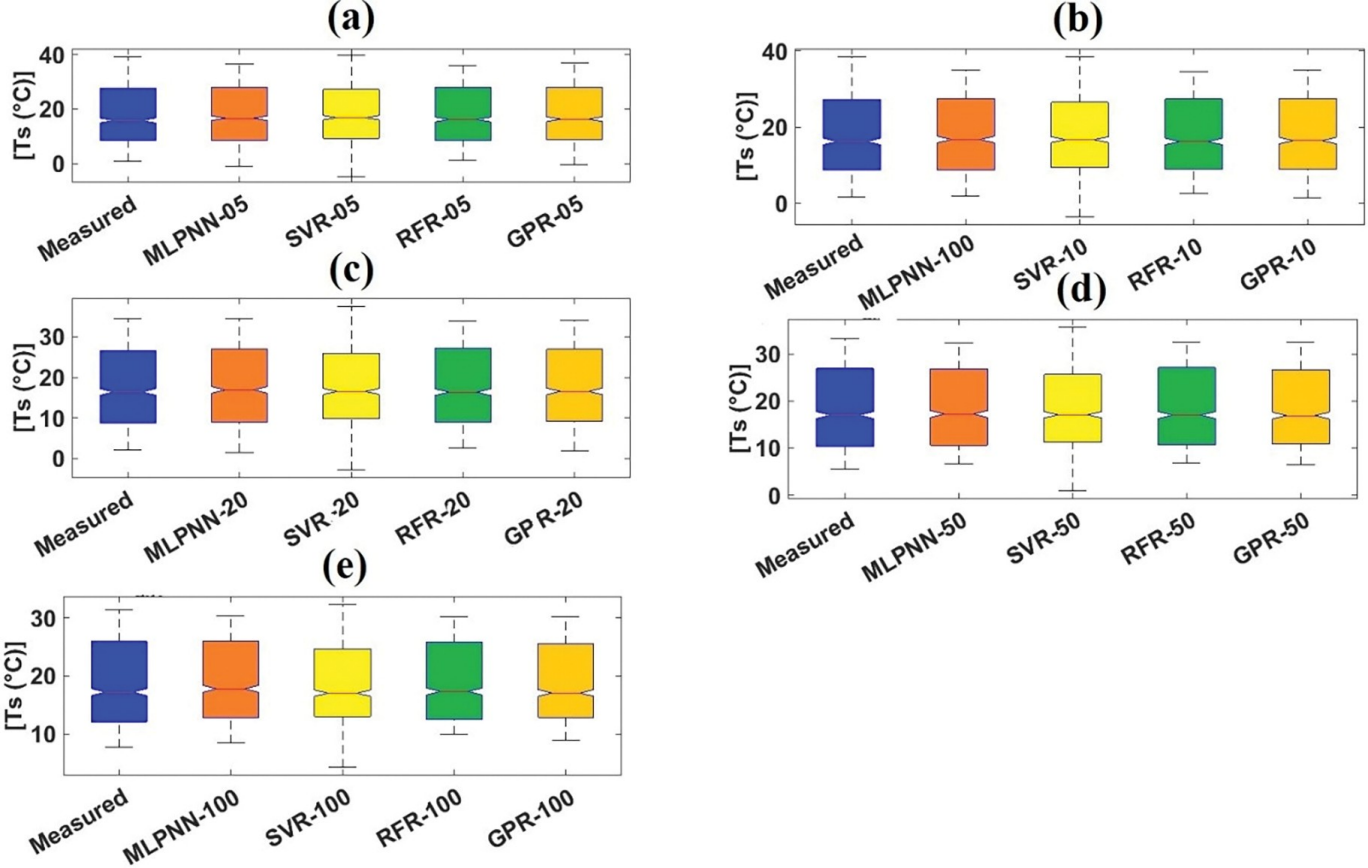
Boxplots of measured and predicted soil temperature of validation dataset for Sulaimani station and different soil depths: (a) 05cm, (b) 10cm, (c) 20cm, (d) 50cm, and (e)100cm.

To validate the predictive efficiency employing the different visual services, boxplot [68], violin plot [69], and spider plot [70] were utilized to highlight the accomplishment of employed models. Boxplot can be defined as a methodology for illustrating the skewness, spread, and locality of predicted values utilizing their quartiles [68,71]. Fig 7(A)–7(E) present the boxplots for employed models with different soil depths from the validation dataset at Sulaimani station. It can be judged from Fig 7(A) that GPR_05 slightly resembled the parameters of boxplot shape (such as lowest value, first quartile, median, third quartile, and highest value) and the length (between top and bottom points) of measured boxplot compared to MLPNN_05, SVR_05, and RFR_05 from the validation dataset. Also, GPR_10 marginally featured the characteristics (i.e., parameters and length) of measured boxplot compared to other ML models with the same soil depth (10cm) from the validation dataset. As well, GPR_20 followed the components of measured boxplot compared to corresponding ML models with identical soil depth (20cm) from the validation dataset. In case of MLPNN_50 and GPR_50, the mentioned ML models matched the essences of measured boxplot compared to SVR_50 and RFR_50 to some extent. Finally, on a small scale, GPR_100 duplicated the various styles of measured boxplot compared to other ML models with equal soil depth from the validation dataset.

**Fig 8:**
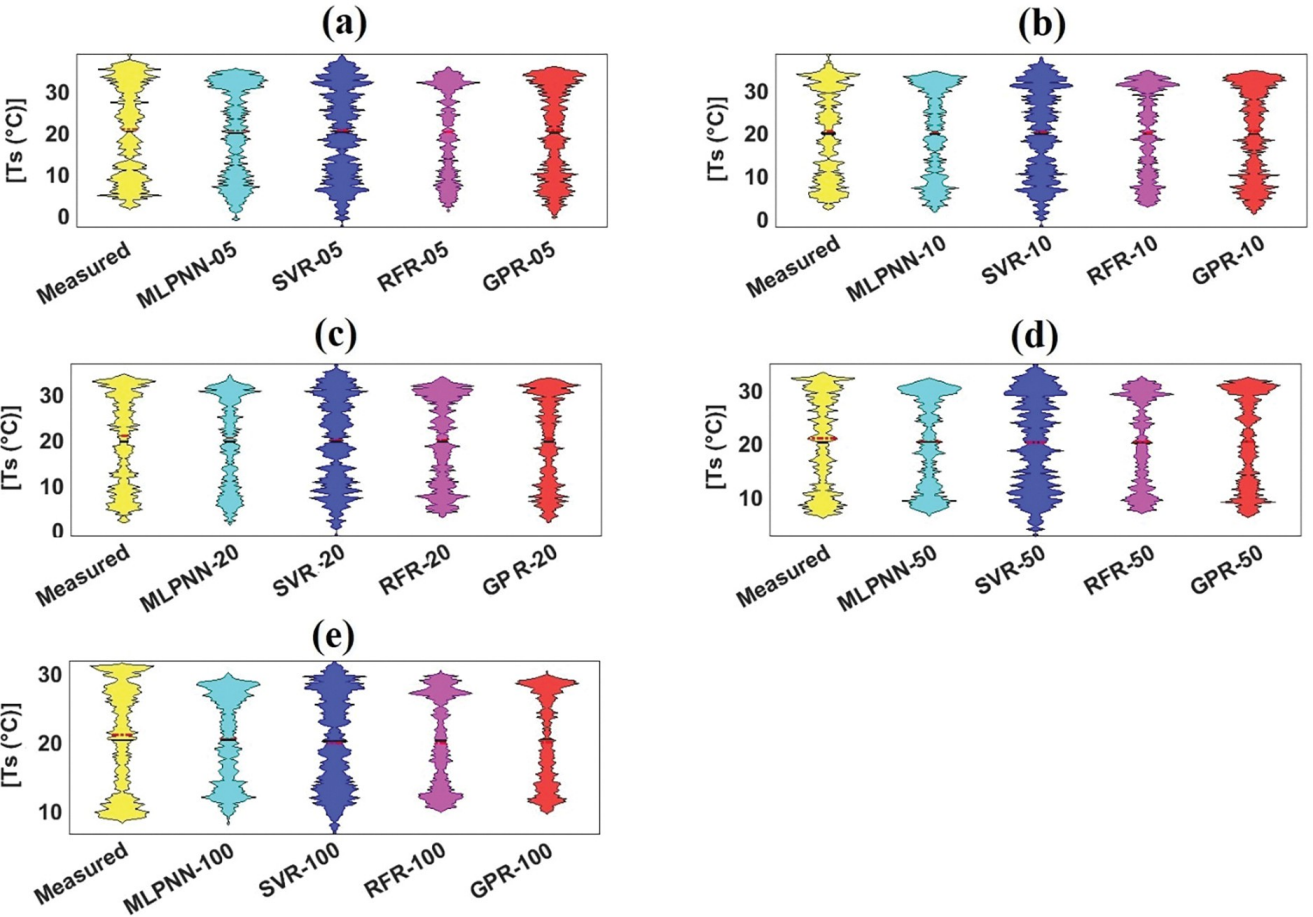
Violin plot of measured and predicted soil temperature of validation dataset for Sulaimani station and different soil depths: (a) 05cm, (b) 10cm, (c) 20cm, (d) 50cm, and (e)100cm.

The violin plot, which underlines the probability spreading of measured and predicted soil temperature with different soil depths, can be arranged as box diagram based on the control of kernel density plot [69]. It can be assessed from Fig 8(A) that GPR_05 stressed the box frame and mentioned values such as mean, median, maximum, and minimum of measured violin plot compared to remaining ML models with same soil depth. Also, considering Fig 8(B), GPR_10 emphasized the form and statistics of measured violin plot compared to MLPNN_10, SVR_10, and RFR_10. In case of 20cm and 50cm soil depths from Fig 8(C) and 8(D), MLPNN and GPR models followed the shape and diverse values of measured violin plot compared to SVR and RFR models. In addition, Fig 8(E) explained that no models could coincide the frame and standards of measured violin plot.

**Fig 9:**
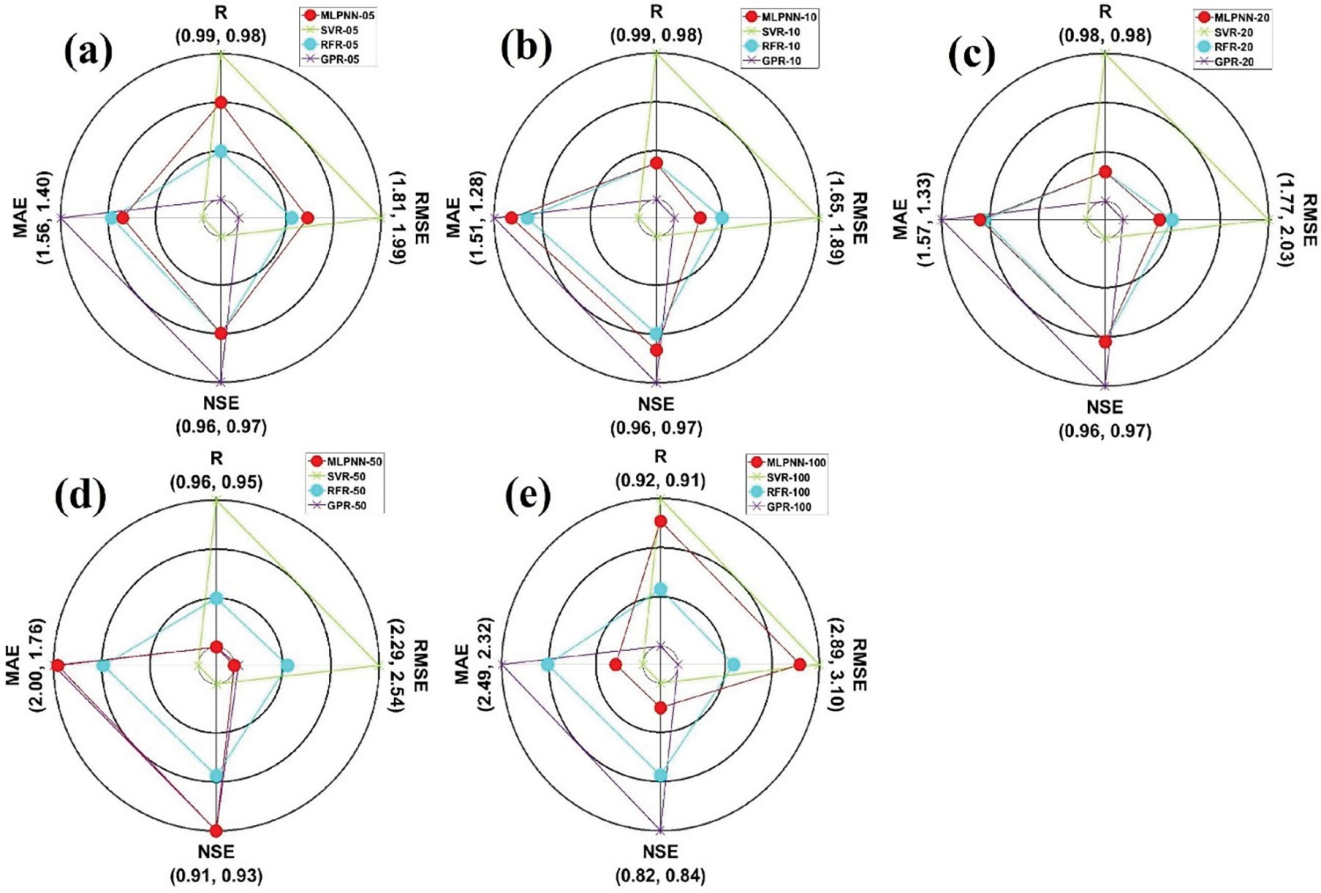
The spider plots showing the models performances of validation dataset for Sulaimani station and different soil depths: (a) 05cm, (b) 10cm, (c) 20cm, (d) 50cm, and (e) 100cm.

A spider plot can be described as a two-dimensional diagram for plotting the values of diverse parameters [70]. In this research, four evaluation indices (i.e., R, NSE, RMSE, and MAE) were allocated on 0, 90, 180, and 270 degrees based on polar coordinate system. It can be evaluated from Fig 9(A)–9(E) that GPR models with diverse soil depths (05, 10, 20, 50, and 100cm) demonstrated the best values compared to other ML models with different soil depths. Also, MLPNN_50 supplied the best output based on the applied ML models with 50cm soil depth.

### 4.2 Prediction of soil temperature based on different soil depths at Dukan station

#### 4.2.1 Application of MLPNN, SVR, RFR, and GRP models

The predictive topics of divergent MLPNN models adopted in this research based on four evaluation indices (i.e., MAE, RMSE, NSE, and R) are organized in Table 4. The predictive values of MLPNN_10 (MAE = 1.110°C, RMSE = 1.481°C, NSE = 0.978, and R = 0.989) were more excellent than those of MLPNN_05, MLPNN_30, and MLPNN_50 from the training dataset. Furthermore, MLPNN_10 (MAE = 1.310°C, RMSE = 1.829°C, NSE = 0.964, and R = 0.982) accomplished more magnificent prediction than MLPNN_05, MLPNN_30, and MLPNN_50 obviously from the validation dataset.

**Table 4:** Performances of different ML models for soil temperature modelling: Dukan station.

	Training				Validation			
Models	R	NSE	RMSE (°C)	MAE (°C)	R	NSE	RMSE (°C)	MAE (°C)
MLPNN_05	0.983	0.966	1.911	1.428	0.976	0.951	2.254	1.678
MLPNN_10	0.989	0.978	1.481	1.110	0.982	0.964	1.829	1.310
MLPNN_30	0.978	0.956	2.019	1.471	0.964	0.927	2.435	1.995
MLPNN_50	0.965	0.932	2.264	1.692	0.937	0.877	2.925	2.325
SVR_05	0.978	0.957	2.146	1.613	0.982	0.964	1.950	1.498
SVR_10	0.986	0.973	1.648	1.222	0.983	0.966	1.766	1.221
SVR_30	0.970	0.940	2.351	1.764	0.967	0.934	2.314	1.874
SVR_50	0.948	0.899	2.750	2.172	0.945	0.892	2.736	2.192
RFR_05	0.989	0.978	1.547	1.135	0.974	0.936	2.587	1.954
RFR_10	0.993	0.985	1.214	0.882	0.976	0.942	2.316	1.708
RFR_30	0.986	0.971	1.635	1.156	0.959	0.906	2.758	2.244
RFR_50	0.977	0.954	1.864	1.387	0.939	0.872	2.983	2.469
GPR_05	0.980	0.960	2.057	1.531	0.980	0.961	2.029	1.531
GPR_10	0.988	0.977	1.532	1.141	0.983	0.967	1.753	1.230
GPR_30	0.975	0.951	2.120	1.553	0.968	0.937	2.270	1.851
GPR_50	0.958	0.918	2.473	1.895	0.949	0.900	2.631	2.120

Among the diverse SVR models, SVR_10 (MAE = 1.222°C, RMSE = 1.648°C, NSE = 0.973, and R = 0.986) provided the first-rate outcomes compared with other ML models from the training dataset. As well, SVR_10 (MAE = 1.221°C, RMSE = 1.766°C, NSE = 0.966, and R = 0.983) produced the outstanding values compared with other ML models from the validation dataset.

Contemplating the particular RFR models, RFR_10 (MAE = 0.882°C, RMSE = 1.214°C, NSE = 0.985, and R = 0.993) yielded the outstanding values compared with RFR_05, RFR_30, and RFR_50 from the training dataset. In addition, RFR_10 (MAE = 1.708°C, RMSE = 2.316°C, NSE = 0.942, and R = 0.976) illustrated the top values compared with RFR_05, RFR_30, and RFR_50 from the validation dataset.

Granting the diverse GPR models, GPR_10 (MAE = 1.141°C, RMSE = 1.532°C, NSE = 0.977, and R = 0.988) furnished the maximal values compared with GPR_05, GPR_30, and GPR_50 from the training dataset. Besides, GPR_10 (MAE = 1.230°C, RMSE = 1.753°C, NSE = 0.967, and R = 0.983) presented the maximum values compared with GPR_05, GPR_30, and GPR_50 from the validation dataset.

Relating the models performance employing training and validation dataset based on RMSE values (°C), SVR_05, SVR_30, and SVR_50 employing validation dataset could outperform the model performance of training dataset. In case of GPR models, the predicted outputs performed by GPR_05 employing validation dataset could surpass the model performance of training dataset. Finally, no MLPNN and RFR models employing validation dataset could exceed model performance of training dataset. Therefore, it can be considered that the model performance of training dataset was better than that of validation dataset at Dukan station.

**Fig 10:**
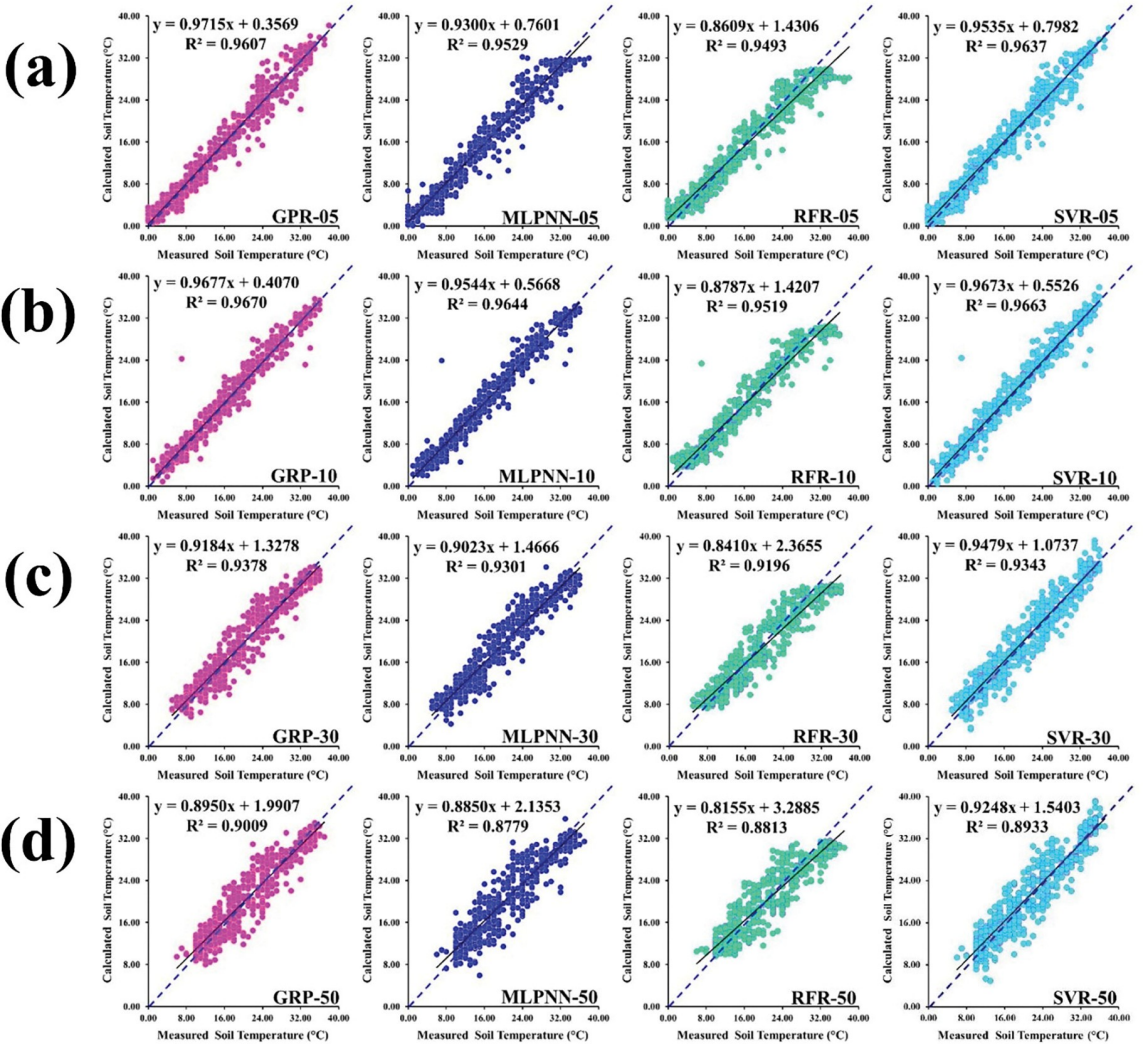
Scatterplot of measured versus predicted soil temperature of validation dataset for Dukan station and different soil depths: (a) 05cm, (b) 10cm, (c) 30cm, and (d) 50 cm.

Fig 10(A)–10(D) emphasize the scatterplot of measured versus predicted soil temperature employing the particular soil depths from the validation dataset at Dukan station. Individual scatterplot includes solid line (fitted), dotted line (equal), optimized regression equation, and determination coefficient, respectively.

Dependent on the values of determination coefficient, GPR_10 (R^2^ = 0.9670) provided the maximal output compared with diverse GPR models including GPR_05, GPR_30, and GPR_50 from the validation dataset. As well, MLPNN_10 (R^2^ = 0.9644) represented the leading output compared with divergent MLPNN models such as MLPNN_05, MLPNN_30, and MLPNN_50 from the validation dataset. Besides, RFR_10 (R^2^ = 0.9519) supported the highest output compared with different RFR models including RFR_05, RFR_30, and RFR_50 from the validation dataset. Furthermore, SVR_10 (R^2^ = 0.9663) supplied the topmost value compared with various SVR models such as SVR_05, SVR_30, and SVR_50 from the validation dataset.

Recognizing on the diverse models with 05cm soil depth, SVR_05 (R^2^ = 0.9637) yielded the best output compared with particular models such as GPR_05, MLPNN_05, and RFR_05 from the validation dataset. Considering 10cm soil depth, GPR_10 (R^2^ = 0.9670) supplied the highest value compared with different models such as MLPNN_10, RFR_10, and SVR_10 from the validation dataset. Fig 10(C) explained that GPR_30 (R^2^ = 0.9378) furnished the topmost output compared with diverse models including MLPNN_30, RFR_30, and SVR_30 from the validation dataset. Based on 50cm soil depth, GPR_50 (R^2^ = 0.9009) gave the top value compared with diverse models including MLPNN_50, RFR_50, and SVR_50 from the validation dataset at Dukan station.

#### 4.2.2 Graphical assistances for performances of machine learning models

**Fig 11:**
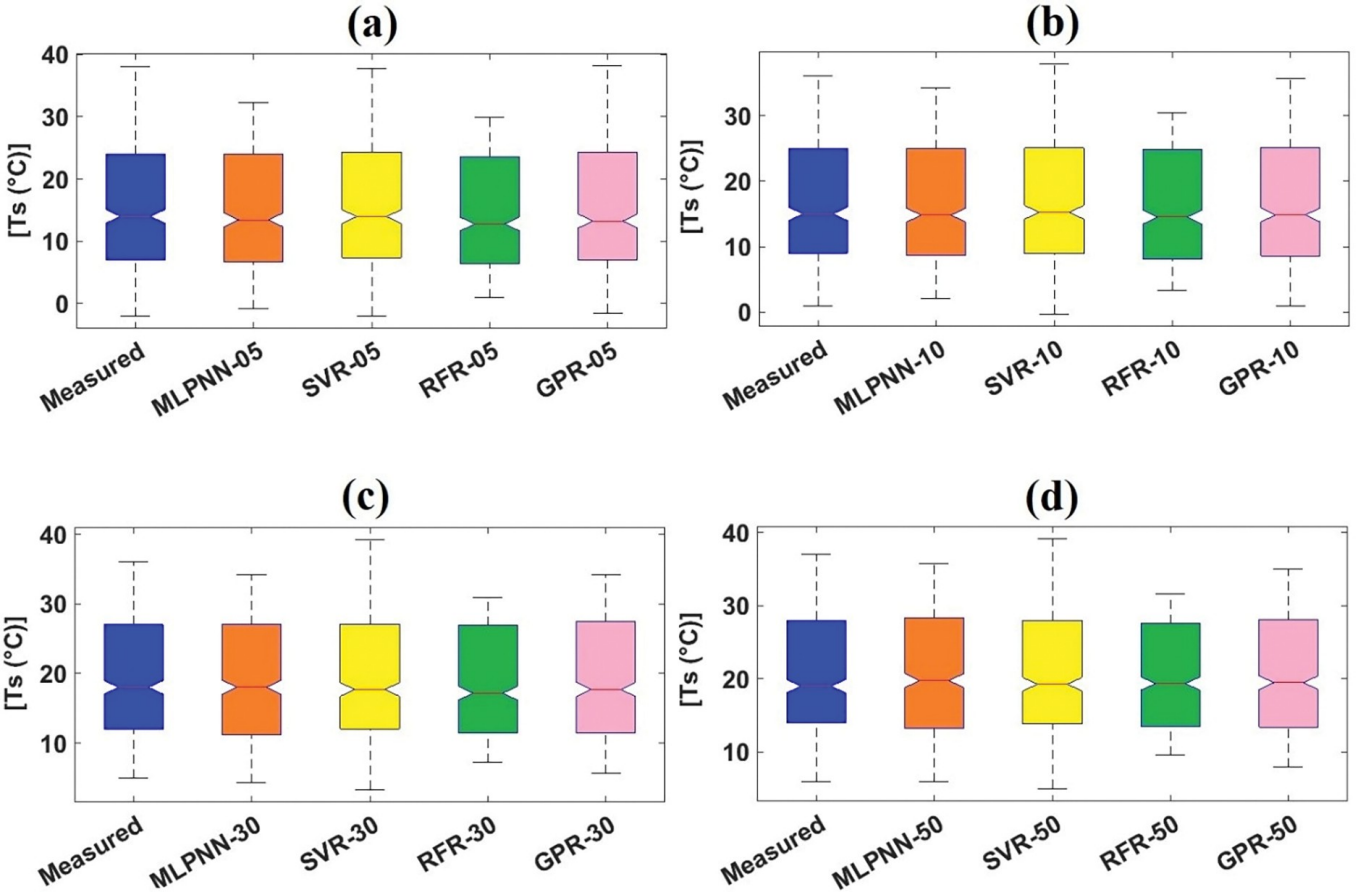
Boxplots of measured and predicted soil temperature of validation dataset for Dukan station and different soil depths: (a) 05cm, (b) 10cm, (c) 30cm, and d) 50cm.

**Fig 12:**
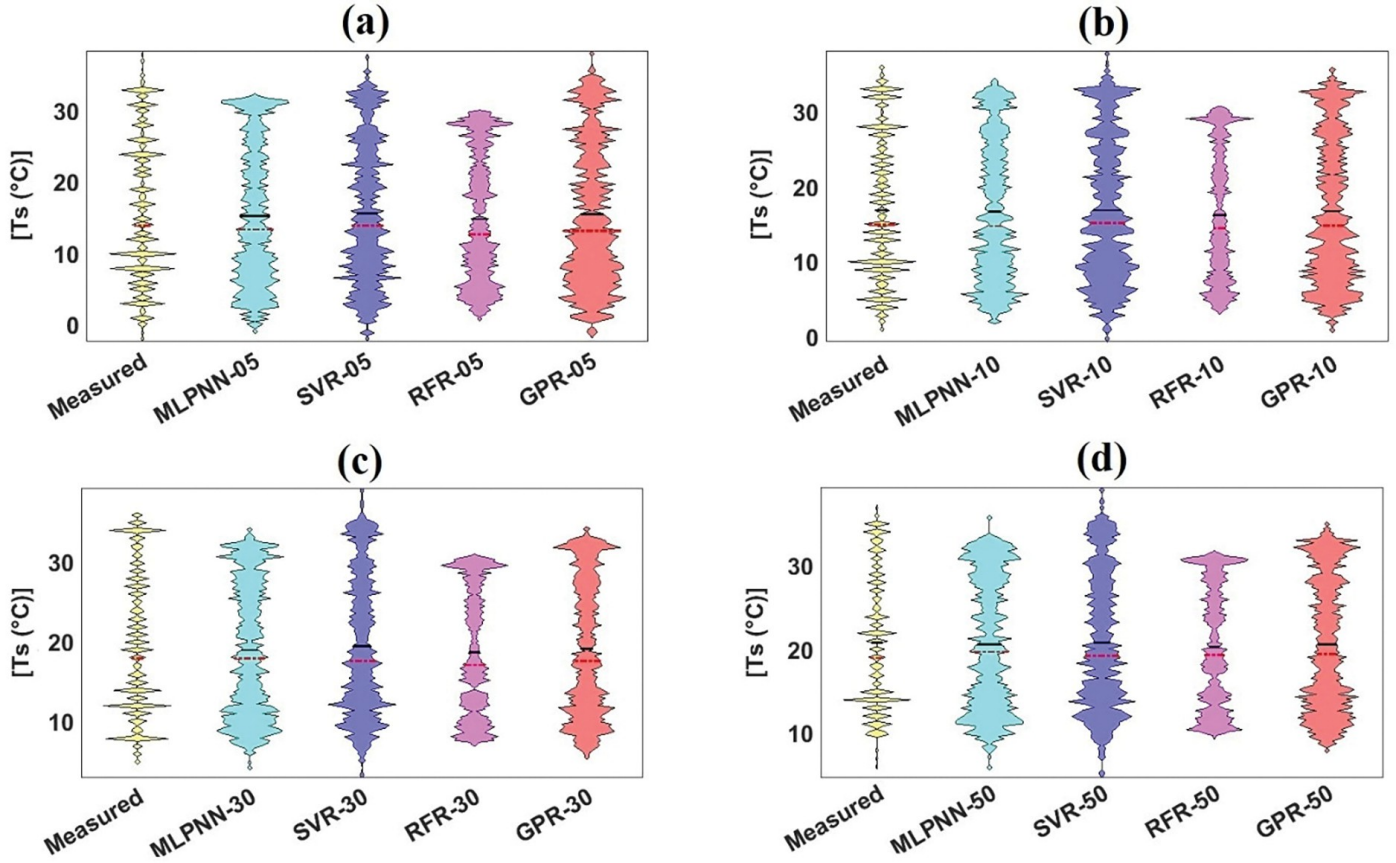
Violin of measured and predicted soil temperature of validation dataset for Dukan station and different soil depths: (a) 05cm, (b) 10cm, (c) 30cm, and (d) 50cm.

**Fig 13:**
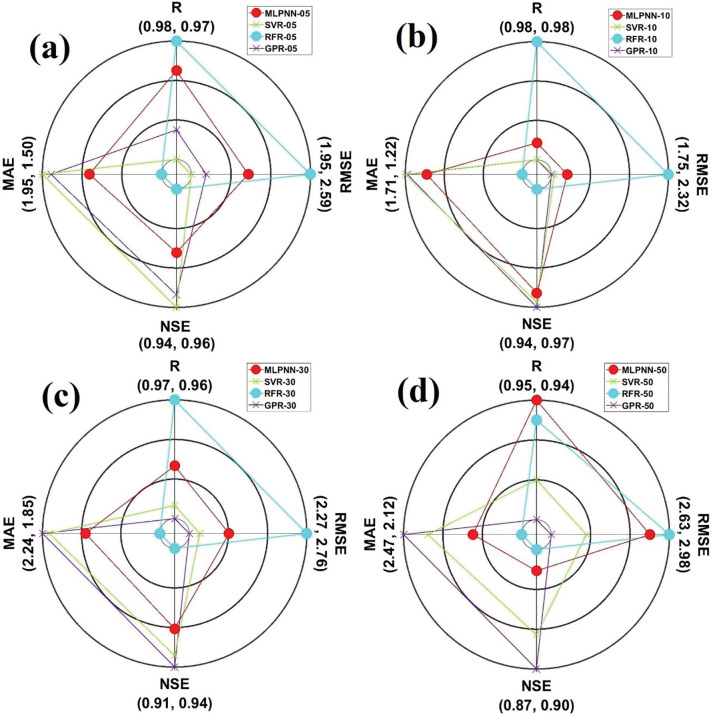
The spider plots showing the models performances of the validation dataset for Dukan station and different soil depths: (a) 05cm, (b) 10cm, (c) 30cm, and (d) 50cm.

Fig 11(A)–11(D) illustrate the boxplots for employed models with diverse soil depths from the validation dataset at Dukan station. It can be assessed from Fig 11(A) that SVR_05 and GPR_05 slightly featured the variables of boxplot shape and the length of measured boxplot compared with MLPNN_05 and RFR_05 from the validation dataset. Besides, GPR_10 slightly followed the characteristics of measured boxplot compared with other ML models (MLPNN_10, SVR_10, and RFR_10) with the same soil depth (10cm) from the validation dataset. Also, GPR_30 matched the components of measured boxplot compared with corresponding ML models (MLPNN_30, SVR_30, and RFR_30) with identical soil depth (30cm) from the validation dataset. In case of GPR_50, the addressed ML models coincided the essences of measured boxplot compared with MLPNN_50, SVR_50, and RFR_50 slightly. Considering violin plots (Fig 12(A)–12(D)), it can be evaluated that no models followed the box frame and diverse values including mean, median, maximum, and minimum of measured violin plots based on all soil depths (05, 10, 30, and 50cm). Regarding the spider plot, it can be resolved from Fig 13(A)–13(D) that GPR models with specific soil depths (10, 30, and 50cm) provided the highest values compared with other ML models with 10, 30, and 50cm soil depths. In case of 05cm soil depth, however, SVR_05 furnished the best output compared with the MLPNN_05, RFR_05, and GPR_05 from the validation dataset at Dukan station.

## 5. Discussion

The present research carried out the predictive ability of soil temperature with the diverse soil depths by employing different ML models at Sulaimani and Dukan stations, Iraq. Based on the values of four statistical indices, the applied ML models with 10cm soil depth provided the best output compared with the corresponding ML models with different soil depths at Sulaimani (05, 20, 50, and 100cm) and Dukan (05, 30, and 50cm) stations.

It is worth to judge that GPR models with all soil depths furnished better efficiency for predicting soil temperature compared to other ML models (MLPNN, RFR, and SVR) with all soil depths except for MLPNN_50 from the validation dataset at Sulaimani station. Furthermore, NSE values covered from 0.842 to 0.974 for GPR models with all soil depths, while the corresponding ranges were demonstrated as 0.822–0.972 (MLPNN), 0.818–0.965 (SVR), and 0.833–0.971 (RFR) from the validation dataset at Sulaimani station.

Also, GPR models with 10, 30, and 50cm soil depths provided better accuracy for predicting soil temperature compared with other ML models based on 10, 30, and 50cm. SVR_05, however, yielded the topmost accuracy for predicting soil temperature compared with MLPNN_05, RFR_05, and GPR_05 from the validation dataset at Dukan station. As well, the field of NSE values was covered from 0.900 to 0.967 for GPR models with all soil depths, whereas the matching fields were provided as 0.877–0.964 (MLPNN), 0.892–0.966 (SVR), and 0.872–0.942 (RFR) from the validation dataset at Dukan station.

Granting the best model based on individual NSE values, GPR_10, which provided the best accuracy, enhanced the predictive efficiency of soil temperature by 0.21% (MLPNN_10), 0.93% (SVR_10), and 0.31% (RFR_10), respectively. Relying on the different soil depths, GPR_10 also boosted the predictive precision of soil temperature by 0.31% (GPR_05), 0.72% (GPR_20), 4.84% (GPR_50), and 15.68% (GPR_100) from the validation dataset at Sulaimani station.

Regarding the topmost model dependent on the specific NSE values, GPR_10 increased the predictive ability of soil temperature by 0.31% (MLPNN_10), 0.10% (SVR_10), and 2.65% (RFR_10), respectively. Dependent on the various soil depths, GPR_10 enhanced the predictive effectiveness of soil temperature by 0.62% (GPR_05), 3.20% (GPR_30), and 7.44% (GPR_50) from the validation dataset at Dukan station.

The comparison of models performance utilizing training and validation dataset demonstrated that the model performance of training dataset was more excellent than that of validation dataset at Sulaimani and Dukan stations clearly. To overcome this phenomenon based on ML models, therefore, the previous researches investigated that model performance utilizing validation dataset which embedded the good quality (e.g., maximum and minimum time series) and abundant quantity (e.g., lots of data available) can provide the outstanding accuracy for prediction issue [72–74].

Contemplating the prior reports and articles for predicting soil temperature utilizing the various soil depths, ML, and DL models, similar investigations have been accomplished. Alizamir et al. (2020) [41] employed the various ML models (ANN, ELM, CART, and GMDH) for predicting monthly soil temperature based on the diverse soil depths, Türkiye. They found that soil temperature with 05, 10, and 15cm soil depths could be predicted utilizing air temperature. In case of soil temperature with 100cm soil depth, additional parameters such as wind speed and solar radiation were required to enhance the prediction of ST. Alizamir et al. (2021) [45] applied a DL (Deep ESN) and three ML (MLPNN, M5Prime, and RF) models for predicting daily ST with the various soil depths, USA. Results explained that a DL model in this study was superior to ML models for predicting daily soil temperature. Bayatvarkeshi et al. (2021) [44] implemented the single (ANN and CANFIS) and hybrid ML models (WANN and WCANFIS) to predict soil temperature, Iran. They indicated that one of hybrid models, WCANFIS, provided the best accuracy for predicting soil temperature. Malik et al. (2022) [47] developed the ML models (SVM, MLP, and ANFIS) combined with the evolutionary algorithms (SMA, PSO, and SHO) for predicting soil temperature in a semi-arid, India. They suggested that SVM-SMA predicted soil temperature better than other models at different soil depths (05, 15, and 30cm).

In this research, since the soil temperature prediction has spotlighted on the few artificial intelligence approaches and soil depths, the current research for predicting soil temperature may be acted as trivial. Thus, the continuous researches by employing different soil depths, ML, and DL models are required to reinforce the predictive accuracy of soil temperature relying on the diverse meteorological parameters. As well, the hybrid approaches for combining the evolutionary algorithm and data preprocess with artificial neural networks are recommended to demonstrate the potential prediction of soil temperature.

## 6. Conclusion

Using an effective modeling tool can serve as a valuable resource for gaining insights into the diurnal and annual fluctuations in ST at various depths. Therefore, this paper proposes several models based on machine learning algorithms to estimate daily ST at two stations in Kurdistan region, Iraq. The models allow analysing accurate soil temperature values as an important factor for calculating the majority of processes occurring within underground ecosystems such as the processes of root development and respiration, control for the conversion and absorption of nutrients by the roots of crops, breakdown of organic matter, and conversion of nitrogen into mineral form in order to assist experts in making informed choices regarding soil health and productivity. Therfore, in developing countries where acquiring data is difficult, application of efficient models that require fewer resources are extremely important. In this study results of medels compared using four evaluation metrics, including correlation coefficient (r), root mean square error (RMSE), Nash-Sutcliffe (NS) efficiency, and mean absolute error (MAE). In terms of RMSE, in Sulaimani station, GPR model produced the most accurate outcomes compared to other approaches at depths of 5 cm (RMSE = 1.814°C), 10 cm (RMSE = 1.652°C), 20 cm (RMSE = 1.773°C), and 100cm (RMSE = 2.891°C). Moreover, The MLPANN exhibited the most superior performance at depth of 50 cm (RMSE = 2.289°C) during the testing phase. Similarly, In Dukan station, GPR model achieved the best results at dephs of 10 cm (RMSE = 1.753°C), 30 cm (RMSE = 2.270°C), and 50 cm (RMSE = 2.631°C). Also, the SVR achieved the best performance at at depth of 5 cm (RMSE = 1.950°C) during the testing phase. Results of this research shows that the suggested method has the potential to estimate daily soil temperature. Accurate predictions of soil temperature can assist in anticipating and comprehending how ecosystems will react to climate change for development a reliable adaptation and mitigation strategies. Additional investigation will place emphasis on employing ensemble-based models, hybrid methodologies, and deep learning algorithms in order to make estimations of daily ST.

## Abbreviations

ANFIS: Adaptive Neuro-Fuzzy Inference System
ANN: Artificial Neural Networks
CANFIS: Co-active Neuro-Fuzzy Inference Systems
CART: Classification and Regression Trees
Deep ESN: Deep Echo State Network
DL: Deep Learning
ELM: Extreme Learning Machine
GMDH: Group Method of Data Handling
GRP: Gaussian Process Regression
M5Prime: M5Prime Tree
ML: Machine Learning
MLP: Multilayer Perceptron
MLPNN: Multilayer Perceptron Neural Network
PSO: Particle Swarm Optimization
RF: Random Forests
RFR: Random Forests Regression
SHO: Spotted Hyena Optimizer
SMA: Slime Mould Algorithm
SVM: Support Vector Machines
SVR: Support Vector Regression
ST: Soil Temperature
WANN: Wavelet transformation combined with ANN
WCANFIS: Wavelet transformation combined with CANFIS
